# A Benzothiazole Derivative (5g) Induces DNA Damage And Potent G2/M Arrest In Cancer Cells

**DOI:** 10.1038/s41598-017-02489-3

**Published:** 2017-05-31

**Authors:** Mahesh Hegde, Supriya V. Vartak, Chandagirikoppal V. Kavitha, Hanumappa Ananda, Doddakunche S. Prasanna, Vidya Gopalakrishnan, Bibha Choudhary, Kanchugarakoppal S. Rangappa, Sathees C. Raghavan

**Affiliations:** 1 Department of Biochemistry, Indian Institute of Science, Bangalore, 560012 India; 2 Department of Studies in Chemistry, University of Mysore, Mysuru, 570006 India; 3 Department of Nanotechnology, Visvesvaraya Technological University, Center for Postgraduate Studies, Bengaluru Region, Muddenahalli, Chikkaballapur, Bangalore, 562101 India; 4 Institute of Bioinformatics and Applied Biotechnology, Electronics City, Bangalore, 560100 India

## Abstract

Chemically synthesized small molecules play important role in anticancer therapy. Several chemical compounds have been reported to damage the DNA, either directly or indirectly slowing down the cancer cell progression by causing a cell cycle arrest. Direct or indirect reactive oxygen species formation causes DNA damage leading to cell cycle arrest and subsequent cell death. Therefore, identification of chemically synthesized compounds with anticancer potential is important. Here we investigate the effect of benzothiazole derivative (5g) for its ability to inhibit cell proliferation in different cancer models. Interestingly, 5g interfered with cell proliferation in both, cell lines and tumor cells leading to significant G2/M arrest. 5g treatment resulted in elevated levels of ROS and subsequently, DNA double-strand breaks (DSBs) explaining observed G2/M arrest. Consistently, we observed deregulation of many cell cycle associated proteins such as CDK1, BCL2 and their phosphorylated form, CyclinB1, CDC25c etc. Besides, 5g treatment led to decreased levels of mitochondrial membrane potential and activation of apoptosis. Interestingly, 5g administration inhibited tumor growth in mice without significant side effects. Thus, our study identifies 5g as a potent biochemical inhibitor to induce G2/M phase arrest of the cell cycle, and demonstrates its anticancer properties both *ex vivo* and *in vivo*.

## Introduction

Cell cycle is an ordered set of events, leading to cell growth and division of the parent cell into two daughter cells^1^. Mammalian cell cycle consists of four coordinated phases, named as G1, S, G2 and mitotic phase which include cell growth, DNA replication, distribution of the duplicated chromosomes to daughter cells and cell division, respectively^2^. Small molecule inhibitors which can affect cell cycle, directly or indirectly are proven to be potent anticancer agents^3^. Direct cell cycle inhibitors act on proteins, which are involved in the process of cell division. On the other hand, indirect cell cycle inhibitors induce specific cell cycle phase arrest due to the damage or inhibition of biomolecules that are related to cell cycle events.

Reactive oxygen species (ROS) are chemically reactive molecules containing oxygen, including, singlet oxygen, superoxide, peroxides and hydroxyl radical. As part of the active cellular metabolism, it is known that physiologically, reactive oxygen species are generated due to the partial reduction of oxygen, which plays a significant role in cell signalling and homeostasis^4^. Reactive oxygen species are lethal to cells due to their ability to damage a variety of cellular macromolecules such as DNA, amino acids, polyunsaturated fatty acids and important cofactors that destabilize the proteins, lipids and necessary enzymes respectively^5^. It is well studied that, cancer cells generate high amount of the reactive oxygen species compared to the normal cells which has been shown to enhance the tumor progression and development^6^. Interestingly, cancer cells also express increased levels of antioxidant proteins to detoxify the extensive ROS, suggesting the requirement of balanced level of intracellular ROS in cancer cells. However, high amount of ROS was also shown to inhibit cancer cell proliferation by arresting cells at G2/M due to extensive DNA damage^7, 8^.

Several small molecule inhibitors are reported in literature to elevate the ROS levels and found to inhibit the cancer cell proliferation. For example, ‘Motexafin gadolinium’, a molecule used to inhibit brain metastasis, is known to inhibit the thioredoxin reductase and ribonucleotide reductase resulting in elevated levels of ROS^9^. ‘Elesclomol’ found to enhance ROS in presence of metal complex like Pt, Ni and Cu was useful in treatment of metastatic melanoma^10^. ‘Buthionine sulfoximine’ reduces the levels of glutathione by inhibiting gamma glutamyl cysteine synthetase which resulted in elevated ROS levels inside the cells and has been used as an adjunct with chemotherapy in the treatment of cancer^11^.

Benzothiazoles are important class of molecules with broad spectrum of biological activities. Certain benzothiazole derivatives are found to effectively inhibit breast cancer cell proliferation^12^. It is known that Benzothiazole derivatives can induce significant amount of ROS production inside the cells causing potent G2/M arrest^13, 14^. Previously, we had reported novel benzothiazole derivatives in which compound named ‘5g’ showed significant anticancer activity^15^. 5g contains a dichloro group on the phenyl ring of arylthiourea at the 2^nd^ and 4^th^ position. In our previous study, we found that compounds possessing electron withdrawing groups at various positions of the phenyl ring of a thiourea moiety displayed significant cytotoxic effect^15^. Interestingly, among the compounds tested, the compound 5g, which possesses chloro substitution at ortho and para positions, was found to be the most potent in cancer cell lines studied. In the present study we show that 5g inhibited growth of breast cancer and leukemic cells but not of PBMCs. Interestingly, we observed that 5g induced distinct arrest at G2/M phase of the cell cycle. Further, we show that 5g reduced mitochondrial membrane potential and induced apoptosis in human leukemic cells. We also found that 5g induced DNA breaks by generating higher levels of intracellular ROS in Nalm6 cells. *In vivo* studies using mouse tumor model showed G2/M arrest in tumor cells leading to tumor regression without exhibiting significant side effects.

## Results

### 5g inhibits growth of various cancer cell lines

**Figure 1:**
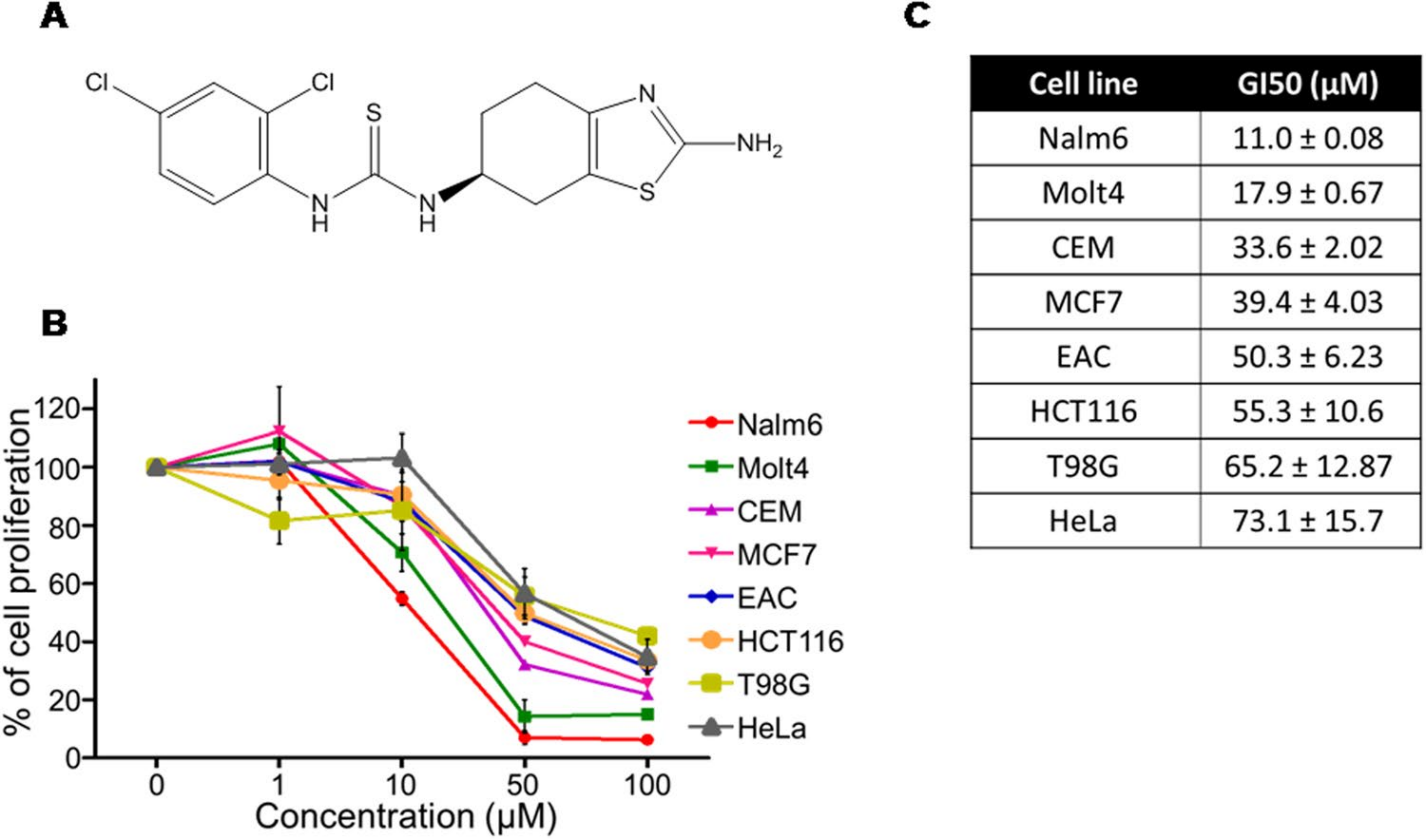
Evaluation of antiproliferative activity of 5g in various cancer cells. (**A**) 2-dimensional structure of 5g. (**B**) Antiproliferative activity of 5g (0, 1, 10, 50 and 100 µM at 48 h) was tested in Nalm6, Molt4, CEM, EAC, HCT116, T98G, MCF7 and HeLa cells using MTT assay. (**C**) Table showing observed GI50 values ± SEM of 5g in various cancer cell lines.

In a previous study, we have reported synthesis, characterization and structure-activity relationship of a series of compounds derived from benzothiazole derivatives^15^. In the present study we have screened a series of cancer cell lines of various origins (Nalm6, Molt4, CEM, MCF7, EAC, T98G, HeLa and HCT116) against the most potent molecule based on previous study (5g) (Fig. 1A). MTT assay results showed that 5g could efficiently inhibit the growth of leukemic cell line Nalm6, followed by Molt4, CEM, MCF7, EAC, HCT116, T98G, and HeLa cells. GI50 was estimated to be 11, 17.9, 33.6, 39.4, 50.3, 55.3, 65.2 and 73.1 µM respectively for these cell lines (at 48 h) (Fig. 1B,C). Since Nalm6 cells exhibited maximum sensitivity towards 5g, it was selected for subsequent *ex vivo* studies.

### 5g induces cell death in leukemic cells more efficiently than in normal cells

**Figure 2:**
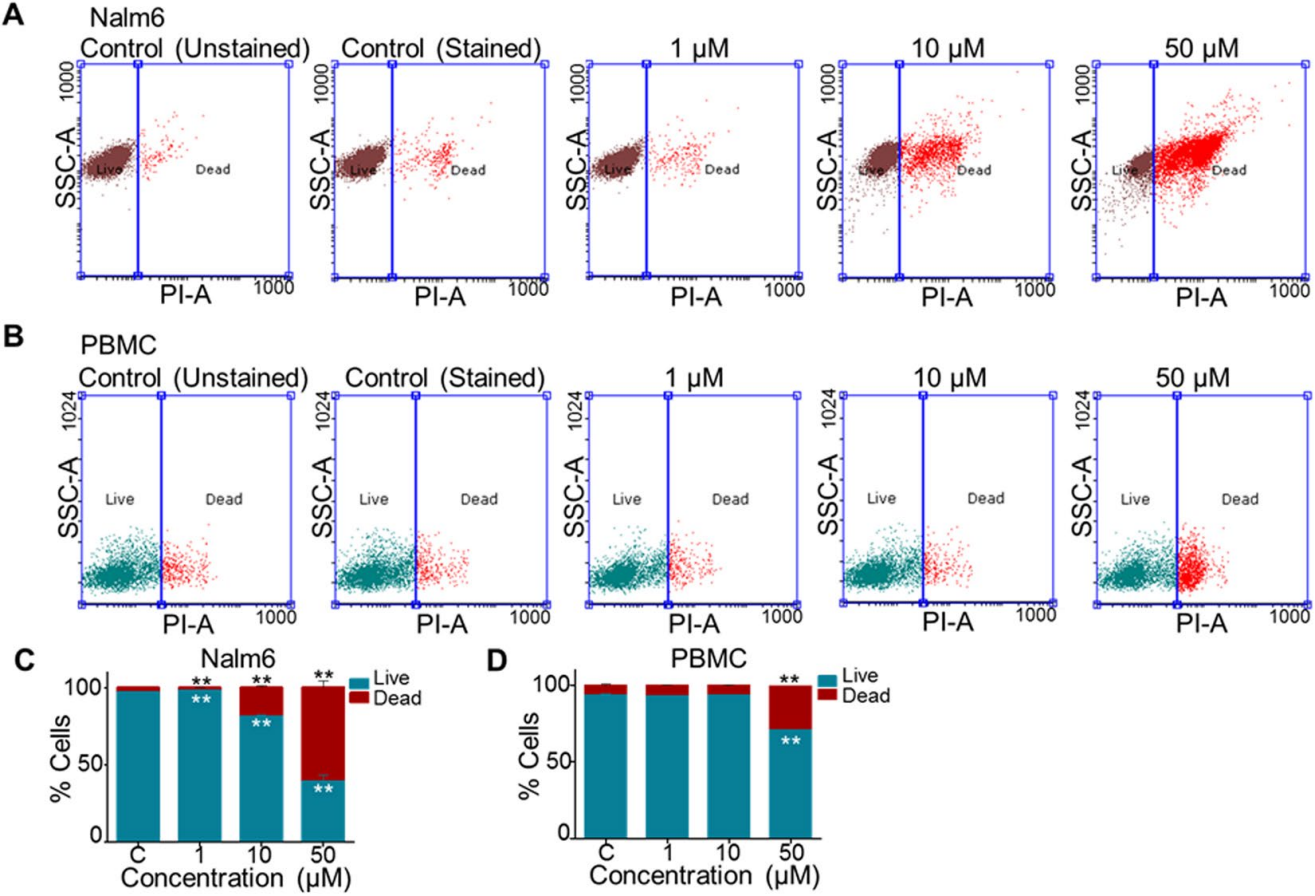
Comparison of cytotoxic effects of 5g in cancer cells and normal cells. (**A**,**B**) Cytotoxic effect of 5g was compared between Nalm6 cells and PBMCs (**B**). Cells treated with 5g (0, 1, 10 and 50 µM; 48 h) were subjected to FACS analysis following staining with Propidium Iodide. Dot plots representing effect of different concentration of 5g on Nalm6 cells (**A**) and PBMCs (**B**). (**C**,**D**) Propidium Iodide positive cells were quantified, plotted as a bar diagram for Nalm6 (**C**) and PBMCs (**D**) respectively (n = 2). Statistical significance was calculated using student t-test and significance was shown if the p-value was equal to or less than 0.05 (*0.05, **0.005, ***0.0005).

Cytotoxic effect of 5g was compared between normal cells and leukemic cells. In order to assess this, PBMCs and Nalm6 cells were treated with increasing concentrations of 5g (0, 1, 10 and 50 µM, 48 h) and cell death was analysed using flow cytometry following staining with Propidium Iodide (PI). Results showed a significant increase in 5g induced cell death in Nalm6 cells (~70% cell death at 50 µM) compared to PBMCs (~25% cell death at 50 µM) (Fig. 2). This observation suggests that 5g could be less toxic in normal cells compared to cancer cells. Effect of 5g treatment in Nalm6 cells was assessed by employing an independent assay, using Calcein-AM and Ethidium homodimer staining. 5g treated (0, 5, 15 and 30 µM; 48 h) Nalm6 cells showed significant positive staining for Ethidium homodimer, while number of Calcein-AM stained positive cells decreased, indicating cell death upon 5g treatment (Suppl. Fig. 1A,B). Further confocal microscopy imaging confirmed the induction of cell death upon treatment with 5g in Nalm6 cells (Suppl. Fig. 1C).

### 5g induces potent G2/M arrest in cancer cells

**Figure 3:**
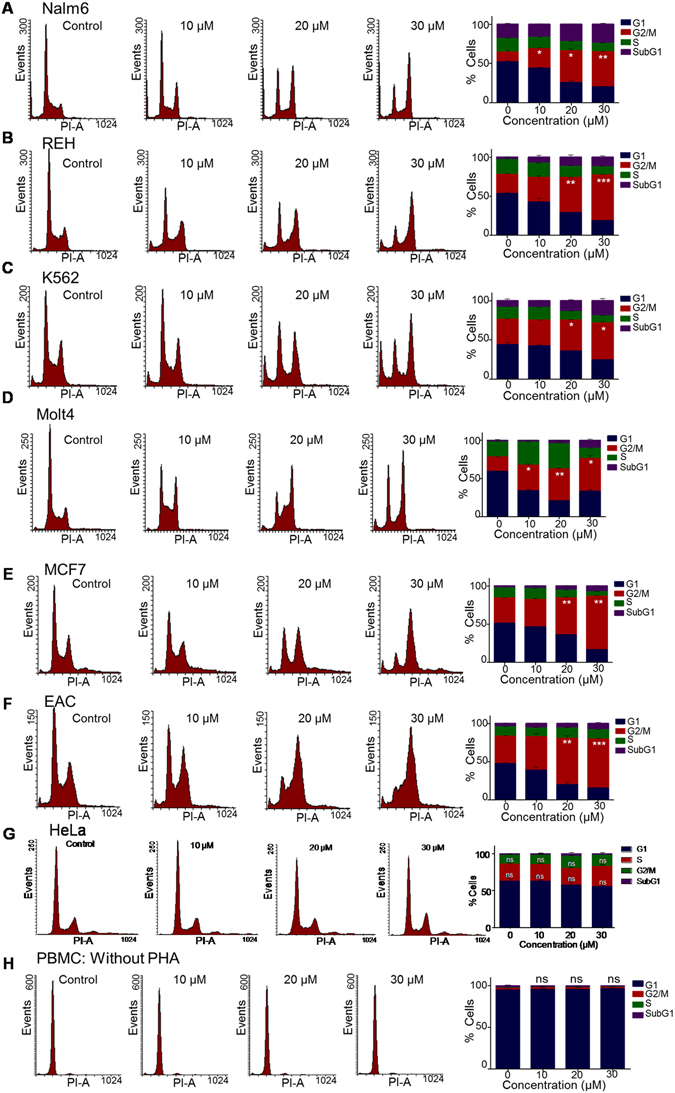
Evaluation of cell cycle arrest in various cancer cell lines following treatment with 5g. Various cancer cells and PBMCs were treated with 5g (0, 10, 20 and 30 µM) for 24 h, stained with PI and cell cycle analysis was carried out using Flow cytometer. (**A**–**H**) Histograms showing cell cycle arrest following treatment with 5g in Nalm6 (**A**), REH (**B**), K562 (**C**), Molt4 (**D**), MCF7 (**E**), EAC (**F**), HeLa (**G**) and PBMC (**H**) cells, respectively. Quantification of different phases of cell cycle was carried out and presented with error bars on the right side. Statistical significance was calculated in G2/M population cells using student t-test and significance was shown if the p-value was equal to or less than 0.05 (*0.05, **0.005, ***0.0005).

The effect of 5g on cell cycle progression was examined in various cancer cells after 24 h of treatment with different concentrations of the inhibitor (0, 10, 20 and 30 µM). Leukemic cell lines (Nalm6, K562, REH, and Molt4), breast cancer cell lines (MCF7 and EAC), cervical carcinoma cell line (HeLa) and normal cells (PBMCs) were used for the study. FACS analysis showed that 5g treatment resulted in significant G2/M arrest in case of almost all the cancer cells tested, except for HeLa, in a concentration-dependent manner (Fig. 3). Interestingly, 5g treated PBMCs did not show significant cell cycle arrest, when tested with the same concentration range (Fig. 3H). Even when the experiment was repeated in presence of PHA (1 µg/ml), a stimulator of cell proliferation, there was no significant cell cycle arrest (Suppl. Fig. 2), suggesting that effect of 5g was minimal in normal cells compared to that on cancer cells. Since we observed a distinct G2/M arrest, further analysis was carried out at various incubation times (12, 36 and 48 h) after treating with lower concentrations of 5g (0, 1, 5, 10 and 15 µM) in Nalm6 cells. Results showed that a concentration as low as 10 µM was sufficient to induce G2/M arrest in Nalm6 cells (Suppl. Fig. 3). We also observed prominent arrest at 36 h time point, however, after 48 h of treatment, cells had either entered SubG1 phase (cell death) or reverted back to normal cell cycle (Suppl. Fig. 3).

**Figure 4:**
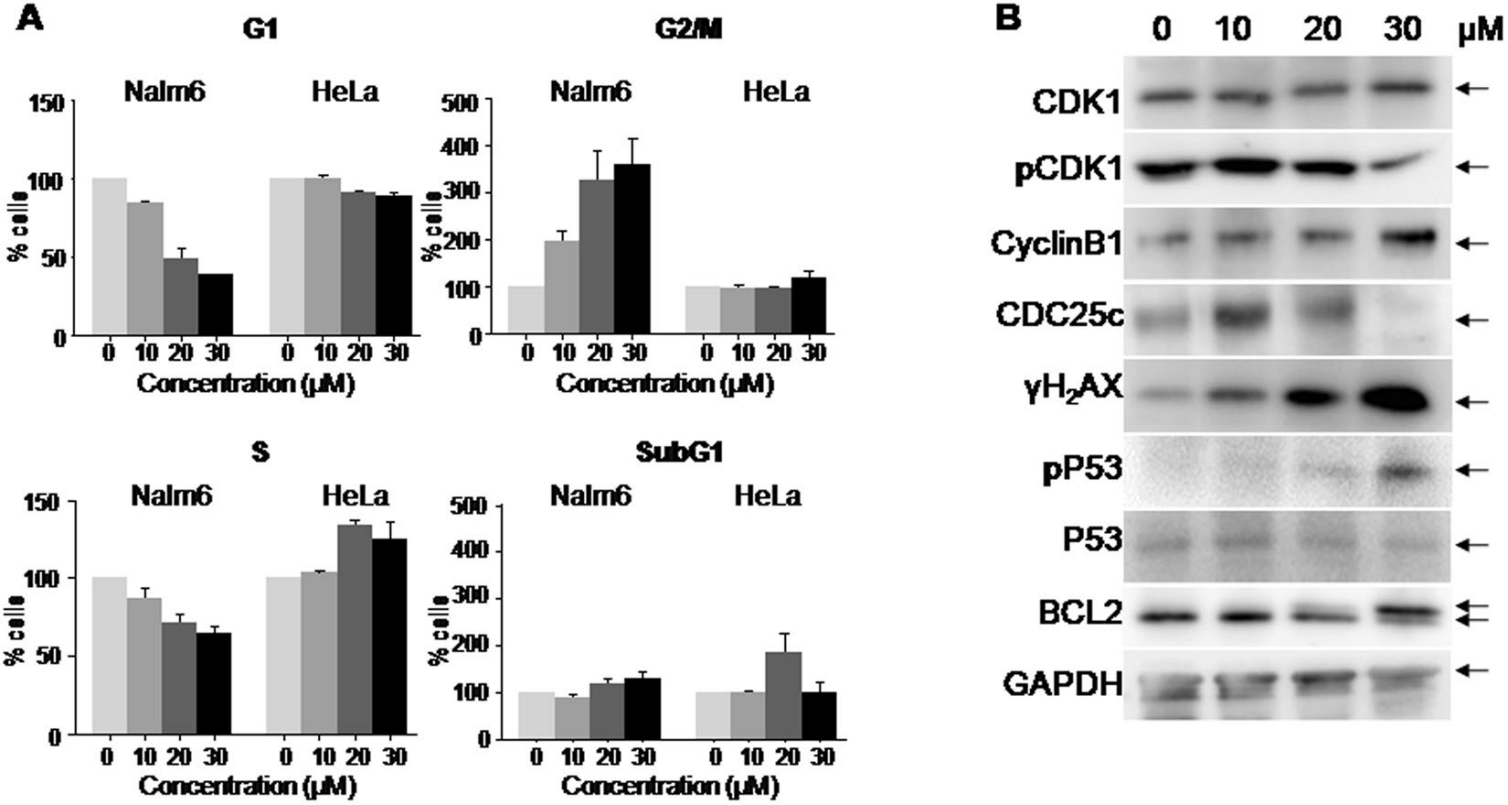
Assessing the effect of 5g on cell cycle progression. (**A**) Histogram depicting quantification of cells in G1, G2/M, S and SubG1 phase of the cell cycle in Nalm6 and HeLa cells following treatment with 5g (0, 10, 20 and 30 µM for 24 h). Each experiment was repeated a minimum of three times, and the data was acquired using flow cytometry. Quantification was performed using Flowing software and the histograms were plotted depicting mean ± SEM (p value: *0.05, **0.005, ***0.0005). (**B**) Western blotting analysis for cell cycle markers following 5g treatment in Nalm6 cells. Cell cycle related proteins, CDK1, pCDK1, CyclinB1, CDC25c, γH2AX, pP53, P53 and BCL2 were analysed using specific antibodies after 5g-treatment (0, 10, 20 and 30 µM; 24 h). GAPDH served as loading control.

Intriguingly, HeLa cells did not exhibit the characteristic G2/M arrest, as observed in other cancer cells upon 5g treatment (Figs 3 and 4A). Cell cycle profiles of 5g treated Nalm6 cells showed accumulation of cells in G2/M phase with a concomitant reduction in the G1 and S phase of the cell cycle, with minor impact on the subG1 phase at 24 h. However, such a cell cycle distribution was not observed in case of HeLa cells upon 5g treatment (Fig. 4A).

Since we observed a robust G2/M arrest by 5g treatment, we explored the possibility of 5g targeting kinases involved in cell cycle regulation. Pharmacophore mapping approach revealed possible binding of 5g with CDK2 (data not shown). CDK2 and CDK1 are highly homologous kinases^16^ and CDK1 function is essential for mitosis^17^. Molecular docking studies using 3D structure of CDK1 and CDK2 indicated that, 5g can bind at the catalytic site of CDK1 as well as CDK2 with binding energies of −10.36 and −9.02, respectively. Importantly, both the binding patterns showed involvement of Leu83 amino acid which is essential for the inhibitory activity of CDK1 and CDK2 proteins^18, 19^. Taken together, these data indicate that 5g may interact with CDK family proteins; however, this needs to be investigated further (Suppl. Fig. 4).

### 5g treatment affects expression of cell cycle related proteins

Further, the protein profile of 5g treated Nalm6 cells was evaluated for potential changes in the cell cycle markers, and DNA damage response proteins, since DNA damage is one of the causes of cell cycle arrest, Nalm6 cells were treated with 5g (0, 10, 20 and 30 µM) and harvested after 24 h and western blotting analysis was performed. Results showed upregulation of CyclinB1 protein, a G2/M phase specific marker (Fig. 4B). Further, significant decrease in phosphorylation status of CDK1 was observed compared to unphosphorylated form (Fig. 4B). Down regulation of CDC25c protein confirmed its role in observed G2/M arrest. Interestingly, we observed a concentration dependent phosphorylation of the antiapoptotic protein BCL2 (Fig. 4B). Several studies have shown phosphorylation of BCL2 occurring in normal G2/M phase of the cell cycle, thus reiterating our finding of a potent G2/M arrest in Nalm6 cells upon 24 h of 5g treatment^20^. Besides, phosphorylation of H2AX and p53 suggested the plausible DNA damage response upon 5g treatment (Fig. 4B).

### 5g induces significant ROS production leading to DNA strand breaks in Nalm6 cells

**Figure 5:**
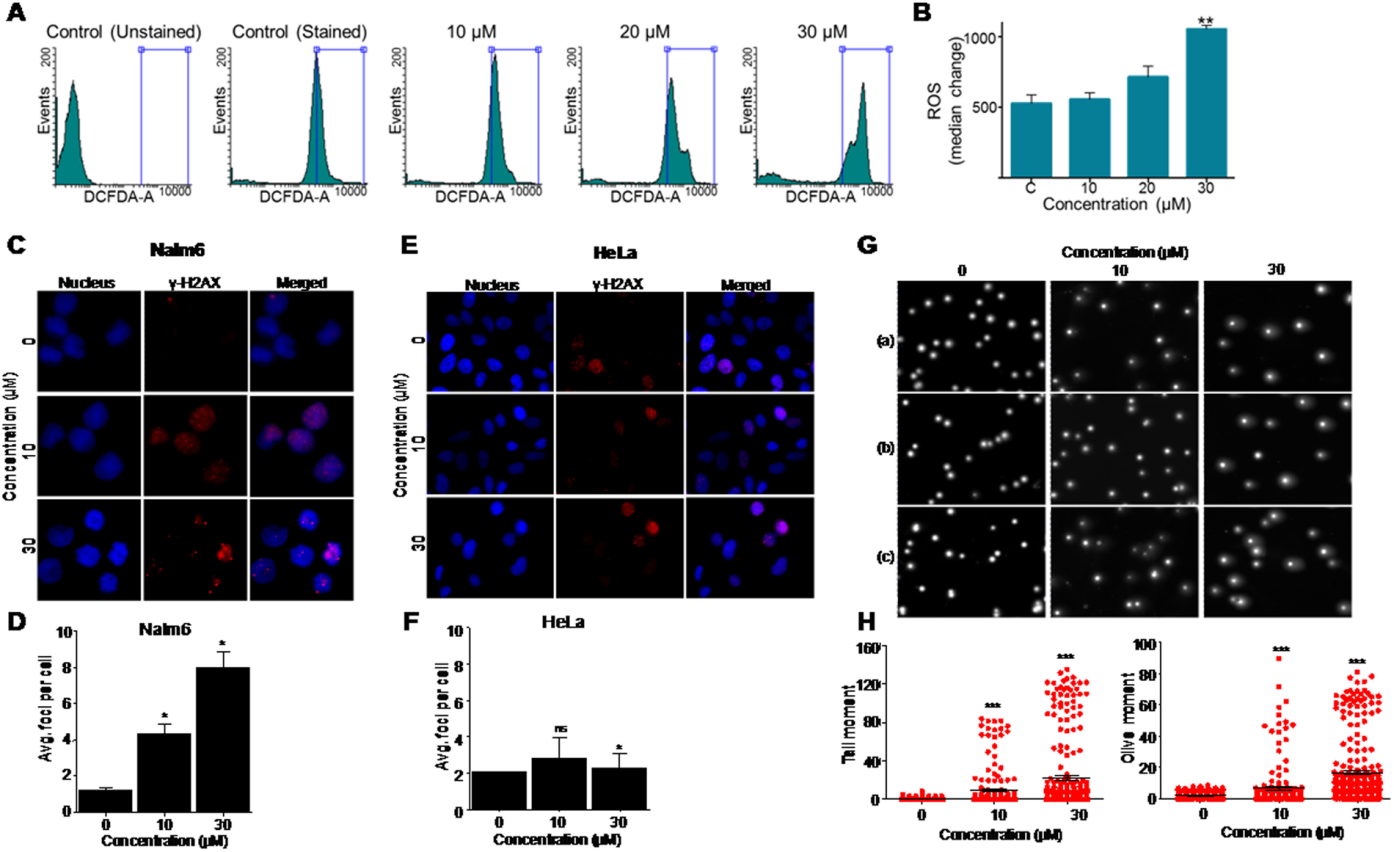
Evaluation of ROS generation and DNA damage within cells following 5g treatment in Nalm6 cells. (**A)** Histogram showing ROS generation after treatment with different concentration of 5g in Nalm6 cells. 5g-treated (0, 10, 20 and 30 µM; 48 h) Nalm6 cells were stained with DCFDA and subjected to FACS analysis. (**B**) Median change with respect to control was quantified and presented as bar diagram with error bars (n = 3). Statistical significance was calculated using student t-test and significance was shown if the p-value was equal to or less than 0.05 (*0.05, **0.005, ***0.0005). (**C**) Representative microscopy images depicting immunofluorescence in Nalm6 cells to detect γ-H2AX foci formation upon treatment with 5g (0, 10, 20 and 30 µM; 24 h). Nucleus is stained using DAPI (blue), γ-H2AX is depicted in red, and the merged image is shown on the right. (**D**) Bar graph depicting average number of foci per cell in Nalm6 cells treated with 5g. A minimum of 100 cells were screened for each sample, foci counted, and plotted as a bar graph showing mean ± SEM (p value: *0.05, **0.005, ***0.0005). (**E**) Representative images showing γ-H2AX staining (red) in HeLa cells upon 24 h of 5g treatment (0, 10, 20 and 30 µM; 24 h). Cells have been counter stained with DAPI to depict nucleus, and merged image is presented at the right side. (**F**) Quantification showing the average number of γ-H2AX foci per cell, plotted as a bar graph depicting mean ± SEM. (**G**) Representative images depicting Nalm6 cells subjected to comet assay analysis post treatment with 5g (0, 10, 20 and 30 µM; 48 h). Cells were stained with propidium Iodide, following neutral comet analysis. Panels a, b and c represent three independent field views from the same sample. (**H**) Scatter plots showing quantification of the extent of DNA double-strand breaks in Nalm6 cells, post 5g treatment. A minimum of 100 cells were analyzed in each case, and Tail Moment (left panel) and Olive Moment (right panel) were calculated by using CometScore software. Each dot represents an individual cell in the quantification panel. All experiments were repeated a minimum of three times, and the error bar plotted as mean ± SEM (p value: *0.05, **0.005, ***0.0005).

To evaluate whether treatment with 5g generates reactive oxygen species inside the cells, DCFDA staining was carried out in treated (0, 10, 20 and 30 µM, 24 h) Nalm6 cells. Interestingly, we observed prominent increase in ROS positive cell population in a concentration dependent manner (Fig. 5A,B). At 30 µM concentration, median of the histogram shifted significantly compared to the control cells, suggesting elevated amount of ROS production upon treatment with 5g.

Since ROS is a known contributor of DNA damage inside cells, we investigated the possibility of DNA double-strand break induction in cells, upon 5g treatment (Fig. 5C–G). γ-H2AX immunofluorescence in Nalm6 cells post 5g treatment (0, 10 and 30 µM for 24 h) showed significant accumulation of γ-H2AX foci in a concentration dependent manner, suggesting induction of DNA damage upon 5g treatment (Fig. 5C,D). Interestingly, HeLa cells did not show significant formation of repair foci at the same concentration and timepoint studied (Fig. 5E,F). Taken together, these observations suggested that induction of DNA damage inside cells, could be one of the mechanisms for the observed G2/M arrest caused by 5g. A direct measure of DNA double-strand break induction inside cells is neutral comet assay analysis of treated cells. Nalm6 cells treated with 5g (0, 10 and 30 µM for 48 h) showed significant amount of DNA in the comet tail region upon electrophoresis, as compared to that in untreated cells (Fig. 5G,H). In addition, a concomitant decrease was observed in the amount of DNA from the comet head, in a concentration dependent manner. This demonstrates that 5g treatment leads to induction of DNA double-strand breaks inside cells (Fig. 5G,H).

### 5g treatment led to reduction in mitochondrial membrane potential and apoptosis induction in leukemic cells

**Figure 6:**
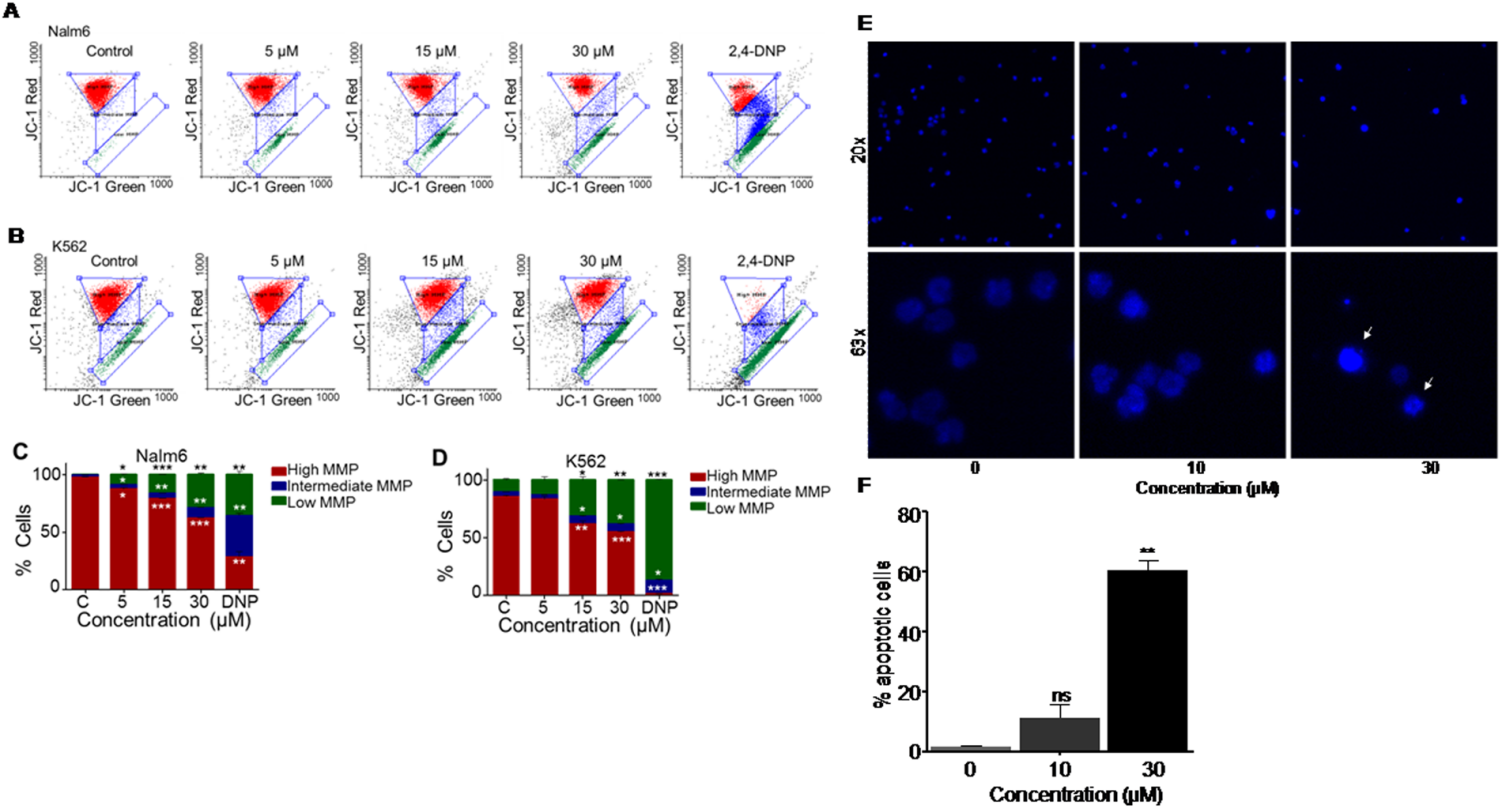
Investigating the mode of cell death induced by 5g in Nalm6 and K562 cells. (**A**) Dot plots representing 5g treated Nalm6 cells stained with JC-1 dye. Cells were treated with 5g (0, 5, 15 and 30 µM) for 48 h and MMP (Mitochondrial Membrane Potential) was measured following staining with JC-1 dye and FACS analysis. (**B**) Dot plots representing 5g treated K562 cells. (**C**,**D**) Bar diagram representing high, intermediate and low mitochondrial membrane potential upon 5g treatment in Nalm6 (**C**) and K562 (**D**) cells, respectively. Statistical significance was calculated using student t-test and significance was shown if the p-value was equal to or less than 0.05 (*0.05, **0.005, ***0.0005). (**E**) Representative microscopy images showing Hoechst stained Nalm6 cells treated with 5g (0, 10 and 30 µM for 48 h). Upper panel represents 20x magnified view of cells, whereas lower panel indicates 63x magnification. Characteristic condensed cells (apoptotic) following 5g (30 µM) treatment are marked by arrowhead. (**F**) Bar graph depicting percentage of apoptotic cells as assessed by Hoechst staining in Nalm6 cells upon 48 h of 5g treatment (0, 10 and 30 µM for 48 h). Each experiment was repeated a minimum of three times, and the bar graph was plotted as mean ± SEM (p value: *0.05, **0.005, ***0.0005).

Mitochondrial membrane potential plays important role in activation of cellular apoptosis. To check the effect of 5g on mitochondrial membrane potential, JC-1 staining was carried out in 5g treated Nalm6 and K562 cells (0, 5, 15 and 30 µM, for 48 h). FACS analysis showed concentration dependent decrease in mitochondrial membrane potential upon treatment with 5g in both Nalm6 and K562 cells (Fig. 6A–D) indicating the role of intrinsic apoptotic pathway following 5g treatment. Further 5g treated Nalm6 cells were assessed for the presence of condensed nuclei, a characteristic feature of apoptotic cells. Cells were treated with 5g (0, 10 and 30 µM, for 48 h) and stained with Hoechst stain to differentiate between healthy and apoptotic cell population (Fig. 6E,F). Interestingly, microscopy analysis showed a significant increase in apoptotic cells upon 5g treatment, wherein particularly 30 µM 5g treated sample exhibited characteristic condensed nuclei stained by Hoechst dye (Fig. 6E,F). Thus 5g treatment resulted in the activation of apoptotic pathway.

### 5g induces apoptosis rather than necrosis

**Figure 7:**
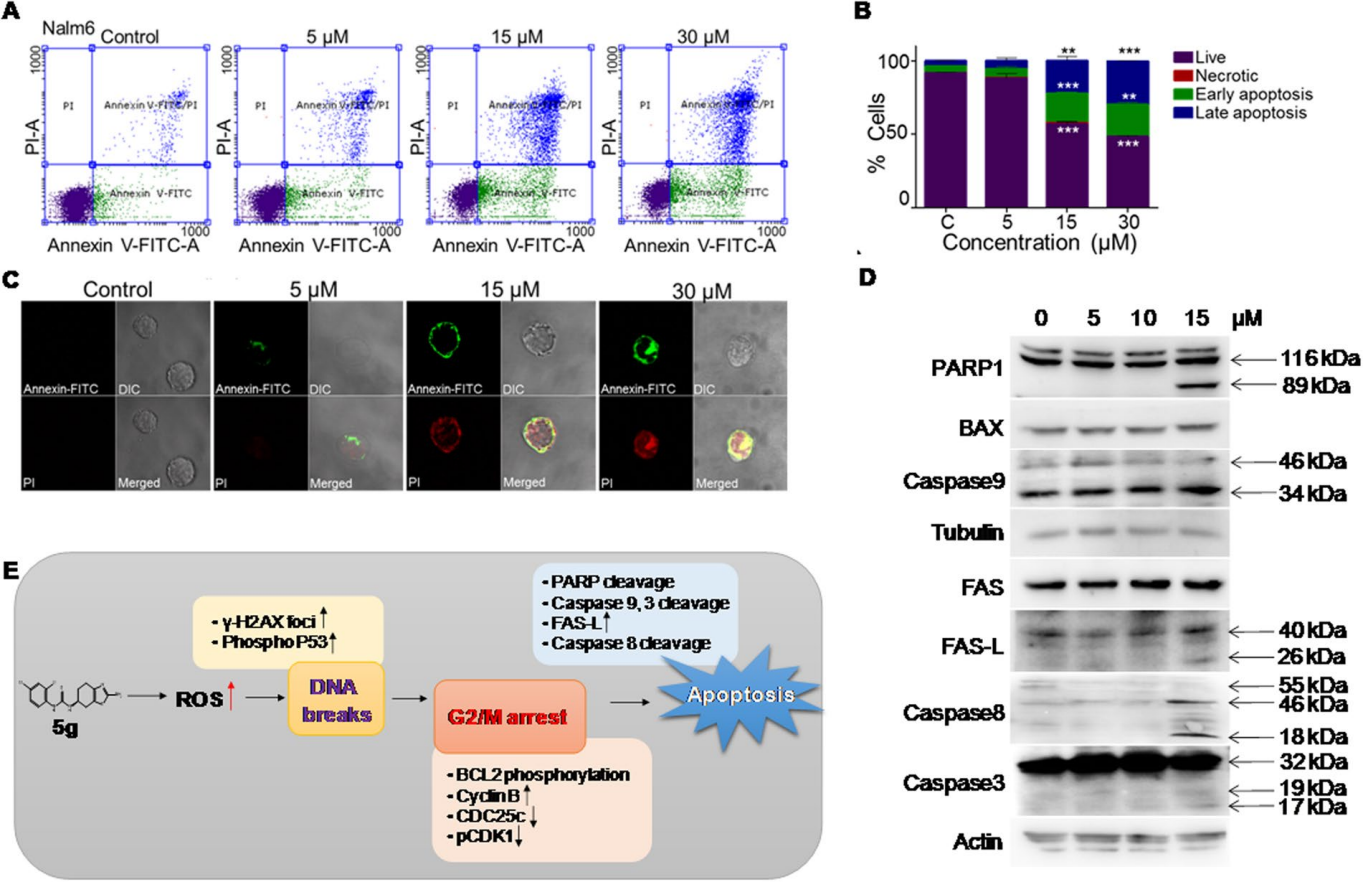
Analysis of mechanism of induction of apoptosis following treatment with 5g in Nalm6 cells. (**A**) Dot plots showing annexin-FITC/PI staining of 5g-treated (0, 5, 15 and 30 µM; 48 h) Nalm6 cells. Following staining, samples were analysed on FACS and images were captured using confocal microscope. 5g-treated Nalm6 cells at different concentration. (**B**) Cells which are positive for annexin-FITC, PI and both annexin-FITC/PI were quantified and represented in bar diagram. (**C**) Confocal microscopy images of 5g-treated cells with 100x magnification. Statistical significance was calculated using student t-test and significance was shown if the p-value was equal to or less than 0.05 (*0.05, **0.005, ***0.0005). (**D**) Western blotting analysis for apoptotic markers following 5g treatment in Nalm6 cells. Apoptotic related proteins were analysed in 5g-treated (0, 5, 10 and 15 µM; 48 h) Nalm6 cells. PARP1, BAX, Caspase9, FAS, FAS-L, Caspase8, Caspase3 specific antibodies were used; Actin served as loading control. (**E**) Model describing the mechanism of action of 5g inside cells. 5g treatment leads to a potent G2/M phase arrest, caused by increased ROS levels and subsequent DNA damage. 5g treated cells eventually undergo cell death by activation of the intrinsic and extrinsic pathway of apoptosis.

To check the mode of 5g induced cells death in Nalm6 cells, annexin-FITC/PI staining was performed. 5g treated (0, 5, 15 and 30 µM, 48 h) Nalm6 cells showed positive staining for annexin-FITC and both annexin-FITC/PI, suggesting presence of both early and late apoptosis upon treatment (Fig. 7A,B). Besides, confocal images showed evidence for both early and late apoptotic cells (Fig. 7C). These results suggested that induction of apoptosis rather than necrosis in 5g treated Nalm6 cells.

Alteration in the expression of apoptotic markers were also tested in 5g treated (0, 5, 10 and 15 µM) Nalm6 cells after 48 h of treatment. Results showed significant level of cleaved PARP1 at 15 µM (Fig. 7D). Besides, upregulation of BAX (proapoptotic protein) and moderate cleavage in Caspase 9 protein were also observed indicative of intrinsic apoptotic pathway. Interestingly, overexpression of FAS and moderate level of Caspase 8 cleavage suggested the involvement of extrinsic apoptotic pathway upon 5g treatment in Nalm6 cells. Further, cleavage of Caspase 3 confirmed the apoptotic induction in Nalm6 cells following treatment with 5g at 48 h time point (Fig. 7D). Taken together, our results demonstrate that 5g induces a potent G2/M phase arrest in the cell cycle, owing to increased levels of ROS, and subsequent DNA damage inside the cell. G2/M arrested cells undergo cell death via the intrinsic or extrinsic pathways of apoptosis (Fig. 7E).

### 5g treatment induces G2/M arrest in tumor cells and decreases the tumor burden *in vivo*

**Figure 8:**
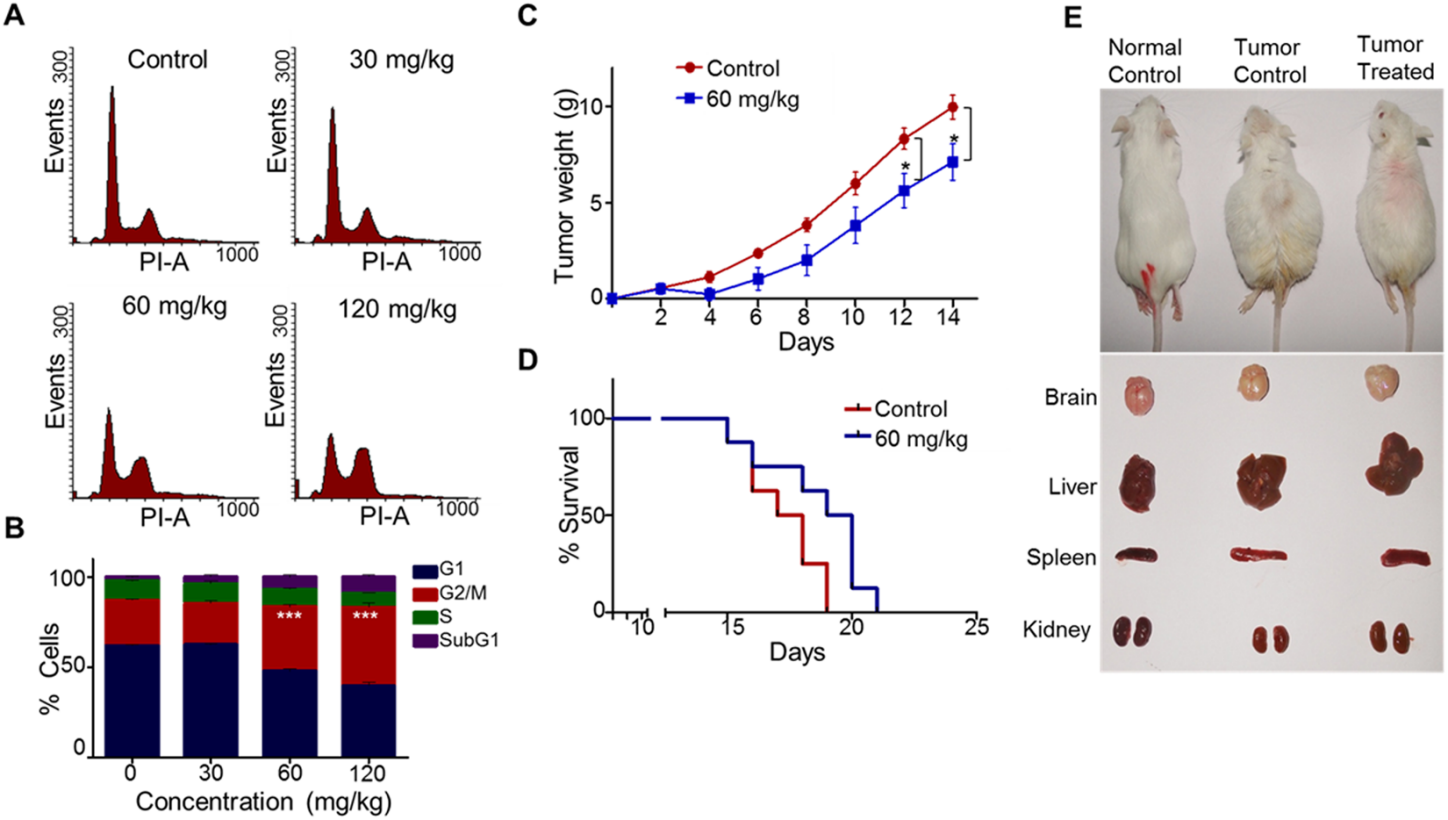
*In vivo* effect of 5 g on EAC tumor model. (**A**,**B**) EAC tumor cells from 5g-administered tumor mice were harvested and processed for cell cycle analysis. Histograms showing effect of 5g on cell cycle progression of EAC tumor cells *in vivo* (A). Quantification of different phases of cell cycle was carried out and presented as bar diagram (B) (n = 4). (**C**–**E**) Effect of 5g on tumor development was tested in EAC tumor model. Body weights of control and 5g (60 mg/kg; 14 doses; one dose/day) treated animals were measured, subtracted with initial body weight of respective animals and presented (n = 8) (**C**). (**D**) Survival graph showing improvement in lifespan after treating with 5g. (**E**) At 15^th^ day, single animal from each group was sacrificed and images were taken, examined for gross changes in organs like brain, liver, spleen and kidney. Statistical significance was calculated in G2/M population cells using student t-test and significance was shown if the p-value was equal to or less than 0.05 (*0.05, **0.005, ***0.0005).

Effect of 5g was investigated in mouse tumor model to ascertain its efficacy *in vivo*. For this, EAC liquid tumor mouse model was used. Following tumor development, different concentrations of 5g was injected (0, 30, 60 and 120 mg/kg; 3 doses continuously; one dose/day), tumor cells were harvested after one day and subjected to cell cycle analysis. Results showed a distinct G2/M arrest in tumor cells following 60 and 120 mg/kg of 5g treatment (Fig. 8A,B).

Efficacy of 5g in decreasing tumor burden was tested in EAC liquid tumor model following treatment with 60 mg/kg body weight of the inhibitor (14 doses continuously; one dose/day). Results showed significant reduction in tumor volume upon administration of 5g in tumor mice (Fig. 8C). Analysis of organs such as brain, liver, spleen and kidney showed normal morphology upon treatment with 5g (Fig. 8E). A moderate increase in life span was also observed following 5g treatment in tumor animals (Fig. 8D).

**Figure 9:**
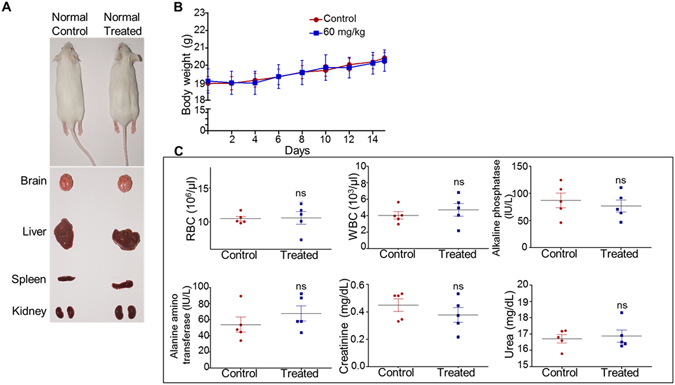
Evaluation of side effect of 5g-treament in mice. Mice were treated with 5g (60 mg/kg; 14 doses, continuously; one dose/day) and examined for potential side effects. (**A**) On 15^th^ day of treatment, control and 5g administered mice were sacrificed and tested for gross changes in organs like brain, liver, spleen and kidney, and images were presented. (**B**) Impact of 5g on body weight in normal mice. The body weight was measured and presented as graph with error bars indicated. (**C**) On 15^th^ day, blood was collected from control and treated mice and evaluated for blood parameters (RBC and WBC count), liver function tests (alkaline phosphatase and alanine amino transferase assay) and kidney function tests (creatinine and urea). Data is presented with error bars (n = 5). Statistical significance was calculated using student t-test and significance was shown if the p-value was equal to or less than 0.05 (*0.05, **0.005, ***0.0005).

Potential side effect of 5g treatment was evaluated in normal mice after treating with 5g (60 mg/kg body weight, 14 doses continuously; one dose/day). No significant changes were observed in 5g treated mice compared to control mice with respect to morphology and over all body weight (Fig. 9A,B). Evaluation of various blood parameters such as WBC, RBC counts did not show any significant difference in cell count (Fig. 9C). Liver and kidney functions assessing various markers such as alkaline phosphatase, alanine amino transferase, creatinine and urea also suggested no/minimal side effect following administration of 5g (Fig. 9C).

## Discussion

Chemical compounds that induce potent cell cycle arrest in cancer cells are helpful in cancer therapeutics^21–25^. Such inhibitors are useful to halt the abnormal cell growth of cancer cells, which would otherwise proliferate at a distinctly faster rate. Inhibition of DNA repair pathways, metabolic pathways, mitotic assemblies and various kinases, which regulate the cell functions have been shown to induce cell cycle arrest in cancer cells.

In the present study mechanism of action of 5g was elucidated. FACS analysis revealed potent G2/M arrest in cells, upon treatment with 5g. Previous studies have reported several characteristic changes in key cell cycle regulators such as CDC25c, CDK2, CyclinB1, etc following G2/M phase arrest^26^. Interestingly, 5g treated Nalm6 cells exhibited downregulation of CDC25c, upregulation of CyclinB1, inactivation of phosphorylated CDK1, phosphorylation of the antiapoptotic protein BCL2, which corroborated with the observed G2/M arrest. Surprisingly, such an arrest was not observed in human PBMCs, suggesting an important role of G2/M phase arrest in mechanism of action of 5g inside cancer cells.

One of the factors that contribute towards G2/M arrest in cells is accumulation of DNA damage^27^. Reactive oxygen species are known to induce various types of DNA damages, particularly DNA double-strand breaks, and that ROS production can induce G2/M arrest through the DNA damage response pathway^7^. Interestingly, our results show significant generation of ROS inside cells upon 5g treatment. Further, induction of DNA double-strand breaks is also observed as assessed by γH2AX foci formation and neutral comet assay. Western blotting results reveal phosphorylation of H2AX and p53, which are markers for DNA damage response. Taken together, these results point towards induction of DNA strand breaks inside cells, and activation of the DNA damage response upon 5g treatment. Surprisingly, 5g failed to induce a G2/M phase arrest in cervical cancer cell line, HeLa. Investigation of γH2AX foci formation in HeLa revealed no significant DNA damage induction following 5g treatment in HeLa, suggesting that DNA damage acts as an important intermediate in the mechanism of 5g action inside cells.

Interestingly, we observed that 5g induced significant cell death in various cancer cell lines. Among the cell lines tested, human leukemic cell line ‘Nalm6’ showed significant sensitivity towards 5g. Importantly, unlike cancer cell lines, cytotoxic effect of 5g was minimal on human PBMCs. Results of various assays suggest that 5g induced cell death in Nalm6 cells by activating apoptosis primarily through intrinsic pathway. However, western blotting studies revealed activation of proteins responsible for extrinsic pathway as well.

Our data revealed increased levels of ROS, and subsequent DNA damage induction upon 5g treatment, leading to the observed G2/M arrest. However, we observed a cell cycle arrest reversal at later timepoints particularly at lower concentrations of 5g treatment. It has been reported that DNA damage induced checkpoints release the cells from arrested phase, once repair of the breaks occurs inside cells, thus reverting the cells back to normal cell cycle progression^27^. Accordingly, at 36 and 48 h post treatment, DNA breaks in the cells could have got repaired by DNA double-strand break repair pathways operating in the cell, resulting in normal cell cycle progression. In contrast, cells that harbour extensive DNA single- and double-strand breaks undergo apoptosis, and are gauged as the subG1 population in FACS analysis^28^. Our results show reversion of cell cycle arrest in lower concentrations of 5g, where cell death proportion is moderate, whereas a potent arrest still persists in case of higher concentrations, with an increased population undergoing apoptosis. Taken together, the results suggest that although 5g induces a potent cell cycle arrest in cells, a subset of cells reverts back to normal cell cycle progression upon repair of DNA breaks, whereas another subset undergoes apoptosis, particularly when concentration of 5g is high.

Although the predominant mechanism of 5g action inside cells was elucidated to be via generation of ROS, subsequent DNA breaks, resulting in a potent G2/M arrest, we also explored the possibility of 5g inhibiting one of the Cyclin Dependent Kinases involved in cell cycle regulation. Docking studies revealed binding potential of 5g to either of the CDKs, suggesting CDK inactivation as the plausible mechanism behind the observed cell cycle arrest, however this aspect warrants further investigation.

*In vivo* tumor studies using 5g showed significant G2/M arrest in treated tumor cells. More importantly there was a significant reduction in tumor burden following 5g treatment in EAC mice. These results suggest that 5g inhibited the tumor cell proliferation significantly. However, complete tumor regression was not observed upon 5g treatment, which was reflected in survival studies of 5g treated EAC mice. Besides, western blotting results showed only moderate cleavage of apoptotic markers like, Caspase 9 and Caspase 3. These results indicate that 5g treatment reduced cancer cell proliferation mainly through G2/M arrest, however complete cell death may not be achieved with 5g treatment alone. Combinatorial therapy strategies involving multiple small molecules need to be explored. However, suitable combinational compounds can be effectively tested when primary target of the 5g compound is established.

One of the most convincing aspects of 5g administration was that it did not cause any adverse effects in normal animals. Consistently it was ‘less toxic’ in e*x vivo* experiments using PBMCs. Interestingly 5g did not cause any cell cycle arrest in normal PBMCs. Therefore, with appropriate combinational compound regimes, 5g could be used effectively in cancer therapeutics. Hence, in the present study, we identify a novel biochemical inhibitor of G2/M phase transition, and provide evidence at proof of principle level that it can be developed as a potent anticancer molecule for chemotherapy.

## Methods

### Chemicals and reagents

All the chemicals used in the present study were of analytical grade and purchased from Sigma (USA). Antibodies were from Santacruz Biotechnology (USA), Calbiochem (USA), Cell Signalling (USA) and Abcam (USA). 5g was synthesized as described previously^15^. DCFDA was purchased from Santacruz Biotechnology (USA).

### Cell culture

Human cancer cell lines, MCF7 (human breast cancer), K562 (chronic myelogenous leukemia), Molt4 (acute lymphoblastic leukemia), CEM (T-cell leukemia), T98G (human glioblastoma multiforme tumor), HeLa (cervical cancer tumor) and EAC (mouse breast cancer) cells were purchased from National Centre for Cell Science, Pune, India and Nalm6 (B-cell leukemia) and REH (B-cell leukemia) cells were kind gifts from Dr. M.R. Lieber, USA. HCT116 cells (colorectal carcinoma) was kind gift from Dr. T R Santhosh Kumar, India. Cells were cultured in RPMI1640/DMEM/MEM (Sera Lab, UK) containing 10% FBS (Gibco BRL, USA), 100 U of Penicillin G/ml and 100 μg of streptomycin/ml (Sigma-Aldrich, USA) at 37 °C in a humidified atmosphere containing 5% CO_2_^29, 30^.

### Growth inhibition studies

The ability of the 5g to inhibit the cancer cell growth was assessed using MTT assay as described previously^31, 32^. Briefly, Nalm6, Molt4 and CEM cells (50,000 cells/ml) were seeded in 24 well plates and treated with different concentration of 5g (0, 1, 10, 50 and 100 µM) for 48 h and cytotoxicity was tested using MTT assay as described before. For EAC, MCF7, HeLa, HCT116 and T98G cells, 5000 cells/well were seeded in 96 well plate; treated with 5g (0, 1, 10, 50 and 100 µM, for 48 h) and MTT assay was carried out as described previously. Experiments were repeated minimum two times and data was presented. Growth inhibitory (GI50) values were determined using GraphPad software prism 5.1 and presented.

### Propidium Iodide live cell staining on PBMCs following 5g-treatment

To check the effect of 5g on normal cells, human peripheral blood mononuclear cells (PBMCs) were used. PBMCs were purchased from Saarum Sciences, Hyderabad, India. PBMCs were cultured in 12 well plates (50,000 cells/ml in RPMI medium with 10% FBS and 1 µg/ml of phytohemagglutinin [PHA]) and incubated with different concentration of 5g (0, 1, 10 and 50 µM) for 48 h. After desired time point, cells were harvested, washed with PBS and stained with Propidium Iodide (50 ng/ml) for 15 min at room temperature. Samples were immediately acquired in BD FACSVerse™ using minimum of 10,000 cells. Similar experiment was carried out in Nalm6 cells and FACS analysis was performed. Data was plotted as dot plot using Flowing software (version 2.5). Bar diagram was presented indicating percentage of Propidium Iodide positive/negative cells (n = 2).

### Assessment of mitochondrial membrane potential

Change in mitochondrial membrane potential following treatment with 5g (0, 5, 15 and 30 µM, 48 h) in Nalm6 and K562 cells (50,000 cells/ml) were tested using JC-1 dye as described earlier and analysed through FACS analysis^33, 34^. A minimum of 10,000 cells were acquired and cells with red and green fluorescence were estimated and plotted as a dot plot. The quantitation was presented as a bar diagram with error bars (n = 2).

### Annexin-FITC/PI staining

Induction of apoptosis in Nalm6 cells upon treatment with 5g was tested through Flow cytometry analysis and confocal microscopy as described earlier^35, 36^. Briefly, Nalm6 cells (50,000 cells/ml) were treated with 5g (0, 5, 15 and 30 µM) for 48 h and stained with annexin-FITC/PI using annexin staining kit (Santacruz, USA); cells were processed for FACS analysis and confocal microscopy as described^35, 36^. DMSO treated cells were used as vehicle control. Experiments were repeated a minimum of two times and data was presented.

### Live dead cell assay using Calcein-AM and Ethidium homodimer staining

Cytotoxic effect of 5g on Nalm6 cells were tested using Calcein-AM and Ethidium homodimer staining through FACS analysis as described earlier^37^. Briefly, Nalm6 cells (50,000 cells/ml) were treated with 0, 5, 15 and 30 µM of 5g for 48 h. Cells were processed and live dead cell assay was performed using flow cytometry. Experiments were repeated a minimum of two times and results were presented as dot plots. Cells which are positive for Calcein-AM or Ethidium homodimer or Calcein-AM and Ethidium homodimer were determined and presented as a bar diagram with error bars.

For confocal microscopic studies, similar treatment regime was considered. Cells were harvested, washed in PBS and incubated with Calcein-AM (1 µM) and Ethidium homodimer (8 µM) for 20 min at 37 °C. Cells were washed again in PBS and fixed using 2% paraformaldehyde for 15 min at room temperature. Further, cells were washed with PBS and mounted on slides using DABCO. Images were captured using inverted confocal laser scanning microscope (Ziess LSM 510 MK4, Germany).

### Cell cycle analysis

Cell cycle analysis was carried out as described previously^38, 39^. Initially, effect of 5g (0, 10, 20 and 30 µM) on Nalm6, REH, K562, Molt4, MCF7 and EAC cells were tested at 24 h time point. Further, Nalm6 cells were treated with 5g (0, 1, 5, 10 and 15 µM) for different time points (12, 36 and 48 h) and cell cycle analysis was carried out. Similarly, effect of 5g (0, 10, 20 and 30 µM) on cell cycle was tested in PBMCs at 24 h time point, cells were processed and analysed through FACS analysis. In all the cases 50,000 cell/ml was seeded and experiments were repeated a minimum of two times and presented as histogram. % of cells in different phases of cell cycle are presented as bar diagram with error bars.

### Assessment of reactive oxygen species in 5g treated Nalm6 cells

To check the generation of reactive oxygen species upon treatment with 5g in Nalm6 cells, DCFDA treatment was carried out as described previously^33^. Nalm6 cells were seeded at a density of 50,000 cells/ml and treated with 5g (0, 10, 20 and 30 µM) for 24 h. Cells were harvested, washed with PBS and stained with 1 µM DCFDA for 15 min at 37 °C and analysed on BD FACSVerse™ with minimum 10,000 cells. DMSO treated cells were used as a vehicle control. Experiments were repeated a minimum of three times and results were presented as dot plots using Flowing software (version 2.5). Total ROS was plotted in bar diagram with respect to median change in control and treated cases with error bars.

### Immunofluorescence

Detection of γ-H2AX foci in Nalm6 and HeLa cells post 5g treatment was performed as described previously^39, 40^. In brief, cells (50,000/ml) were seeded on coverslips and treated with 5g (10 and 30 µM for 24 h), followed by harvesting and fixing of the cells using 4% paraformaldehyde for 10′ at room temperature. Fixed cells were then permeabilized using 0.1% Triton x100 for 5′, followed by blocking (1 h at 4 °C). After blocking, cells were probed with appropriate primary antibody, followed by Alexa flour secondary, and counter stained using the nuclear marker DAPI. Images were acquired using Apotome Fluorescence Microscope (Zeiss, Germany). A minimum of 100 cells were imaged per sample, and analyzed for repair foci formation in control and treated cases. Each experiment was repeated three times and the quantification was plotted as a bar graph showing mean ± SEM.

### Comet assay

In order to gauge the extent of DNA double-strand breaks induced post 5g treatment in Nalm6 cells, a neutral comet assay was performed as described before^29, 40^. In brief, Nalm6 cells (50,000/ml) were seeded in 6 well plates, treated with 5g for a period of 48 h, harvested and washed with PBS. Cells were then mixed with low melting agarose at 37 °C and spread onto agarose-coated glass slides. After solidification of the agarose-cells mixture, the slides were submerged in neutral lysis buffer overnight at 37 °C. Slides were then washed with neutral running buffer, followed by electrophoresis at 12 V for 25′. Staining was performed using Propidium Iodide (10 µg/ml) for 20′ at room temperature, followed by image acquiring in Apotome Fluorescence microscope (Zeiss, Germany). A minimum of 200 cells were imaged for each sample, followed by analysis using the CometScorev1.5 software. Tail moment and Olive moment parameters were analyzed and plotted as scatter plots showing mean ± SEM.

### Western blot analysis

Expression of apoptotic and cell cycle related proteins was analysed in Nalm6 cells following 5g treatment by western blot analysis as described previously^41^. To check the expression profile of apoptotic related proteins, Nalm6 cells (50,000 cells/ml) were treated with 5g (0, 5, 10 and 15 µM) for 48 h; to check the expression profile of cell cycle related proteins, Nalm6 cells (50,000 cells/ml) were treated with 5 g (0, 10, 20 and 30 µM) for 24 h; at the end of the time point, cells were harvested and processed for western blot analysis as described previously. Primary antibodies used in this study were; PARP1 was from Calbiochem (USA); BCL2, BAX, Caspase9, Caspase8, Caspase3, Tubulin, FAS, CDC25c, P53, pP53, CyclinB1 and GAPDH were from Santacruz (USA); γH2AX (from Abcam, USA); CDK1, pCDK1 and actin were from Cell Signalling (USA). Membranes were developed using chemiluminescent reagents (Millipore, USA) and images were acquired using gel documentation system (LAS 3000, Fuji, Japan). Tubulin, Actin and GAPDH were served as loading control. Western blotting was carried out minimum two times and representative images were presented.

### Hoechst staining

In order to assay induction of apoptosis in 5g treated Nalm6 cells, Hoechst staining was performed as described before^42^. Briefly, Nalm6 cells (50,000/ml) were seeded in 6 well plates and treated with increasing concentrations of 5g (10 and 30 µM) for a period of 48 h. Cells were harvested and washed with PBS, stained with Hoechst (1 µg/ml) for 10′ at room temperature and imaged using a Zeiss Apotome Fluorescence Microscope (Zeiss, Germany). A minimum of 100 cells were imaged and analyzed for each sample, and characteristic condensed cells were counted and presented as a bar graph showing mean ± SEM.

### *In vivo* studies

All the animal experiments were carried out according to the protocols approved from the animal ethical committee of Indian Institute of Science (IISc), Bangalore, India (CAF/Ethics/289/2012). Swiss albino mice, 6–8 weeks old, weighing 18–22g were purchased and maintained in the central animal facility, Indian Institute of Science (IISc), Bangalore, India. The animals were housed in polypropylene cages and provided with standard pellet diet (21% protein, 5% lipids, 4% crude fiber, 8% ash, 1% calcium, 0.6% phosphorus, 3.4% glucose, 2% vitamin, and 55% nitrogen-free extract [carbohydrates]) and water ad libitum. The mice were maintained under controlled conditions of temperature and humidity with a 12 h light/dark cycle.

*In vivo* efficacy of 5g was tested using EAC tumor model in Swiss albino mice as described previously with minor modifications^43^. EAC tumor was developed inside the intraperitoneal cavity of the mice (liquid tumor model). Briefly, 2.5 × 10^5^ EAC cells from the donor mice were injected into the intraperitoneal cavity of the recipient mice and were used for the experiment. In EAC tumor model, two different parameters were analysed; ability of the 5g to induce G2/M arrest *in vivo* and ability of the 5g to regress the tumor development.

5g was properly emulsified using 1% methyl cellulose (nearly 500 µl/10 mg of 5g) and dissolved in double distilled water. Throughout the animal experiment, 5g was administered using intraperitoneal injection and control animals received the similar treatment with the solution containing double distilled water and methyl cellulose (without 5g).

Initially, ability of the 5g to induce G2/M arrest *in vivo* was assessed using different concentration of 5g. After eight days of EAC injection, three doses of 5g were given (0, 30, 60 and 120 mg/kg of animal body weight; one dose/day) continuously and after 24 h of final dose, EAC cells from the control as well as treated mice were collected, processed for cell cycle analysis as described earlier (n = 4).

To check the ability of 5g to regress the tumor development, EAC tumor cells were injected in Swiss albino mice and 5g was injected (60 mg/kg; one dose/day) from the day one of tumor injection till 14^th^ day continuously. Body weight of the animals was measured throughout the experiment to check the tumor development and presented in the final graph after subtracting with initial body weight of the animals (n = 8). Body weight of normal Swiss albino mice without tumor was assessed for evaluating normal growth of the mice. At 15^th^ day, one animal from each group was sacrificed and gross appearances of the organs were imaged.

### Evaluation of side effect of 5g treatment in mouse

To test the side effect of 5 g in normal mice, Swiss albino mice were taken and 5g was administered for 14 days continuously (60 mg/kg; one dose/day). Throughout the experiment, animal body weight was measured and plotted to check the difference in control and 5g treated mice. 24 h after the final dose, all the animals were sacrificed, blood was collected and processed to check the side effect as described previously^44^. Blood parameter test (RBC and WBC), liver function (alanine amino transferase and alkaline phosphatase) and kidney function tests (urea and creatinine) were carried out using specific kits from Span diagnostic limited, India and presented (n = 5) and gross appearance of the organs were imaged.

### Docking studies with Cyclin Dependent Kinases

Molecular docking studies were carried out using Autodock 4.2 program (PMID: 19399780). Briefly, 3D structure of CDK1 and CDK2 were retrieved from protein data bank (PDB ID: 5HQ0 and 2VTP, respectively). Ligand was removed and PDBQT files were generated for protein as well as 5g. Grid files were generated using the appropriate dimensions (5HQ0: 30.84, −69.63, 186.64 for X, Y and Z dimensions, respectively; 2VTP: 28.68, 5.31 and 60.28 for X, Y and Z dimensions, respectively). Rigid docking was carried out using Lamarckian genetic algorithm (GA run was kept 10 with medium search parameter). Best pose was selected according to lower binding energy and presented in the final image. Images were created with PyMOL (http://www.pymol.org/).

### Statistical analysis

The error bars were expressed as average ± SEM. Significance was calculated using student t-test after comparing each value with respective controls using GraphPad software prism 5.1. The values were considered as statistically significant, if the p-value was equal to or less than 0.05 (*0.05, **0.005, ***0.0005).

## Electronic supplementary material

Supplementary information
