# Supplementary material for: A Benzothiazole Derivative (5g) Induces DNA Damage And Potent G2/M Arrest In Cancer Cells
Source: Sci Rep. 2017 May 31;7:2533. doi: 10.1038/s41598-017-02489-3 (PMC5451441; doi:10.1038/s41598-017-02489-3)
Supplement: Supplementary file 1 — Supplementary information [file 41598_2017_2489_MOESM1_ESM.pdf]

# A BENZOTHIAZOLE DERIVATIVE (5g) INDUCES DNA DAMAGE AND POTENT G2/M ARREST IN CANCER CELLS

Mahesh Hegde[a, b*], Supriya V. Vartak[a*], Chandagirikoppal V. Kavitha[a], Ananda Hanumappa[a], Doddakunche S. Prasanna[b], Vidya Gopalakrishnan[a,c], Bibha Choudhary[c], Kanchugarakoppal S. Rangappa[b] and Sathees C. Raghavan[a**]

From [a]Department of Biochemistry, Indian Institute of Science, Bangalore 560012, India, [b]Department of Studies in Chemistry, University of Mysore, Mysuru 570006, India, [c]Institute of Bioinformatics and Applied Biotechnology, Electronics City, Bangalore 560100, India

*Equal first authors

**To whom correspondence should be addressed

Sathees C. Raghavan :Ph. +91 80 22932674; Fax: +91 80 23600814;

e-mail: sathees@biochem.iisc.ernet.in

Key words: Cell cycle, G2/M arrest, Apoptosis, Inhibitor, DNA damage, CDK, ROS, Cancer therapy

## Supplementary Figure legends

**Supplementary Figure 1. Live dead cell assay of 5g-treated Nalm6 cells.** Nalm6 cells were treated with 5g (0, 5, 15 and 30 µM; 48 h), stained with Calcein-AM and Ethidium Homodimer following which samples were subjected to FACS and confocal microscopy. **A-C.** Dot plots showing 5g-treated Nalm6 cells (A). Cells which are positive for Calcein-AM, Ethidium homodimer and both Calcein-AM/Ethidium Homodimer were quantified and represented in bar diagram (B). The staining was further confirmed using confocal microscopy and representative images are presented with 100x magnification (C). Statistical significance was calculated using student t-test and significance was shown if the p-value was equal to or less than 0.05 (0.05*, 0.005**, 0.0005***).

**Supplementary Figure 2. Cell cycle analysis of 5g treated PBMCs.** PBMCs were treated with different concentrations of 5g (0, 10, 20 and 30 µM; 24 h) in presence of PHA and subjected to FACS analysis. In each case, quantification of different phases of cell cycle was carried out and presented as bar diagram (n=2). Statistical significance was calculated in G2/M population cells using student t-test and significance was shown if the p-value was equal to or less than 0.05 (0.05*, 0.005**, 0.0005***).

**Supplementary Figure 3. Cell cycle analysis following treatment with 5g in Nalm6 cells, after different time points.** 5g treated Nalm6 cells (0, 1, 5, 10 and 15 µM) were harvested at different time points and processed for cell cycle analysis. **A-C.** Histograms showing effect of 5g on Nalm6 cells at 12, 36 and 48 h after treatment. Quantification of different phase of cell cycle was carried out and presented as bar diagram for respective time points (n=2). Statistical significance was calculated in G2/M population cells using student t-test and significance was shown if the p-value was equal to or less than 0.05 (0.05*, 0.005**, 0.0005***).

**Supplementary Figure 4. Molecular docking studies to assess the binding potential of 5g to Cyclin Dependent Kinases (CDK1 and CDK2).** Docked poses of 5g with two of its potential

targets (based on higher docking scores), CDK1 (A) and CDK2 (B). Original ligand LZ9 was shown in ‘cyan’ and docked pose of 5g was shown in red colour. Image depicting important amino acid interactions with 5g is also presented as a separate panel below.

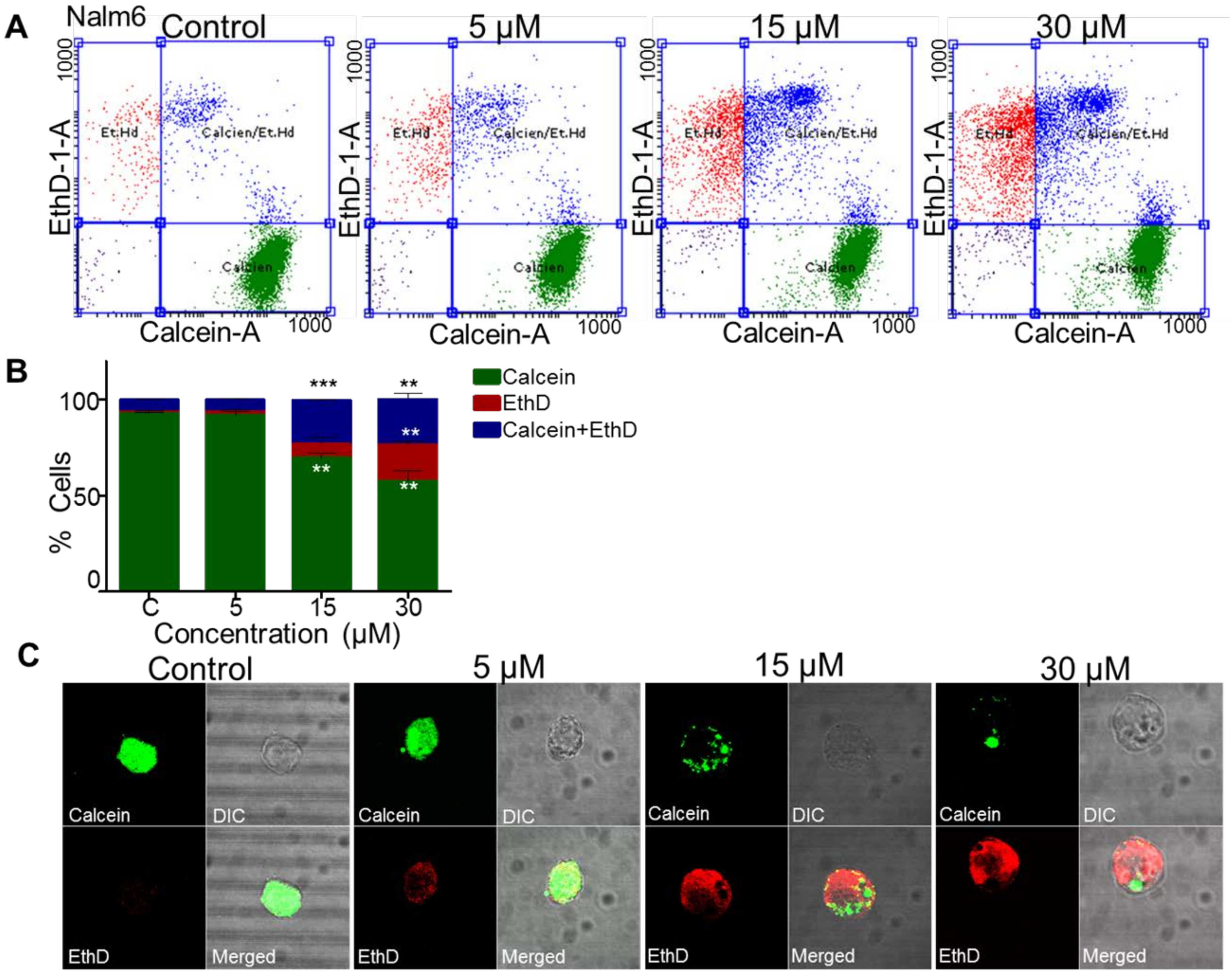


Suppl. Figure 1

PBMC: With PHA

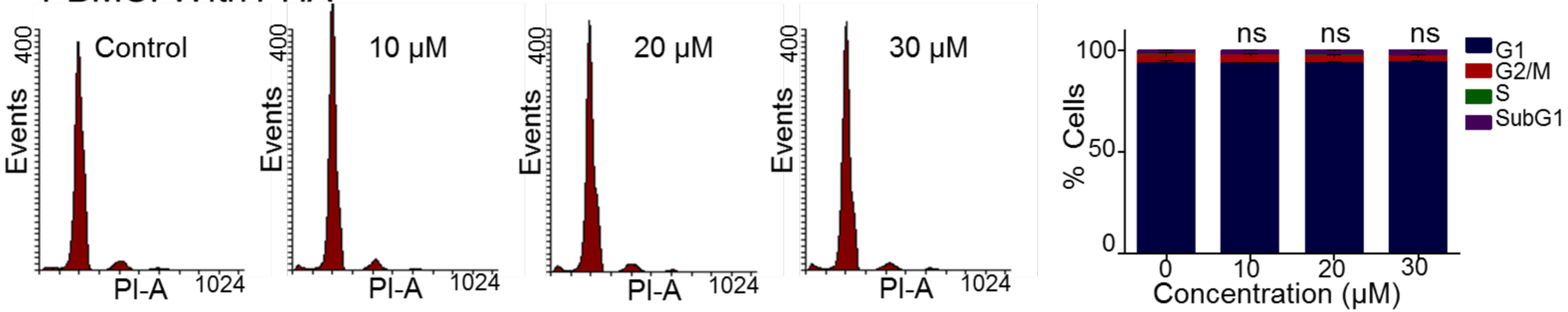


Suppl. Figure 2

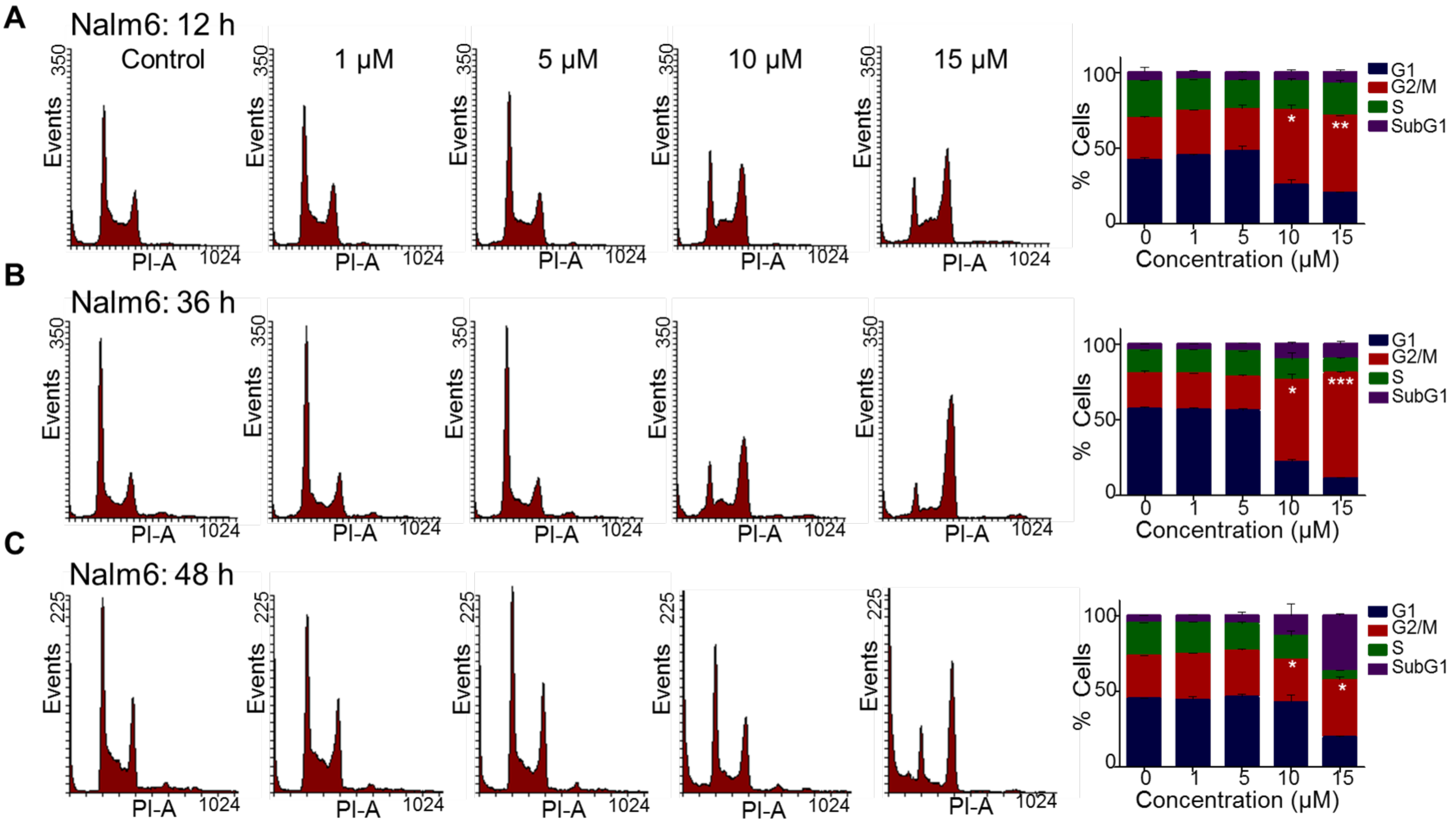


Suppl. Figure 3

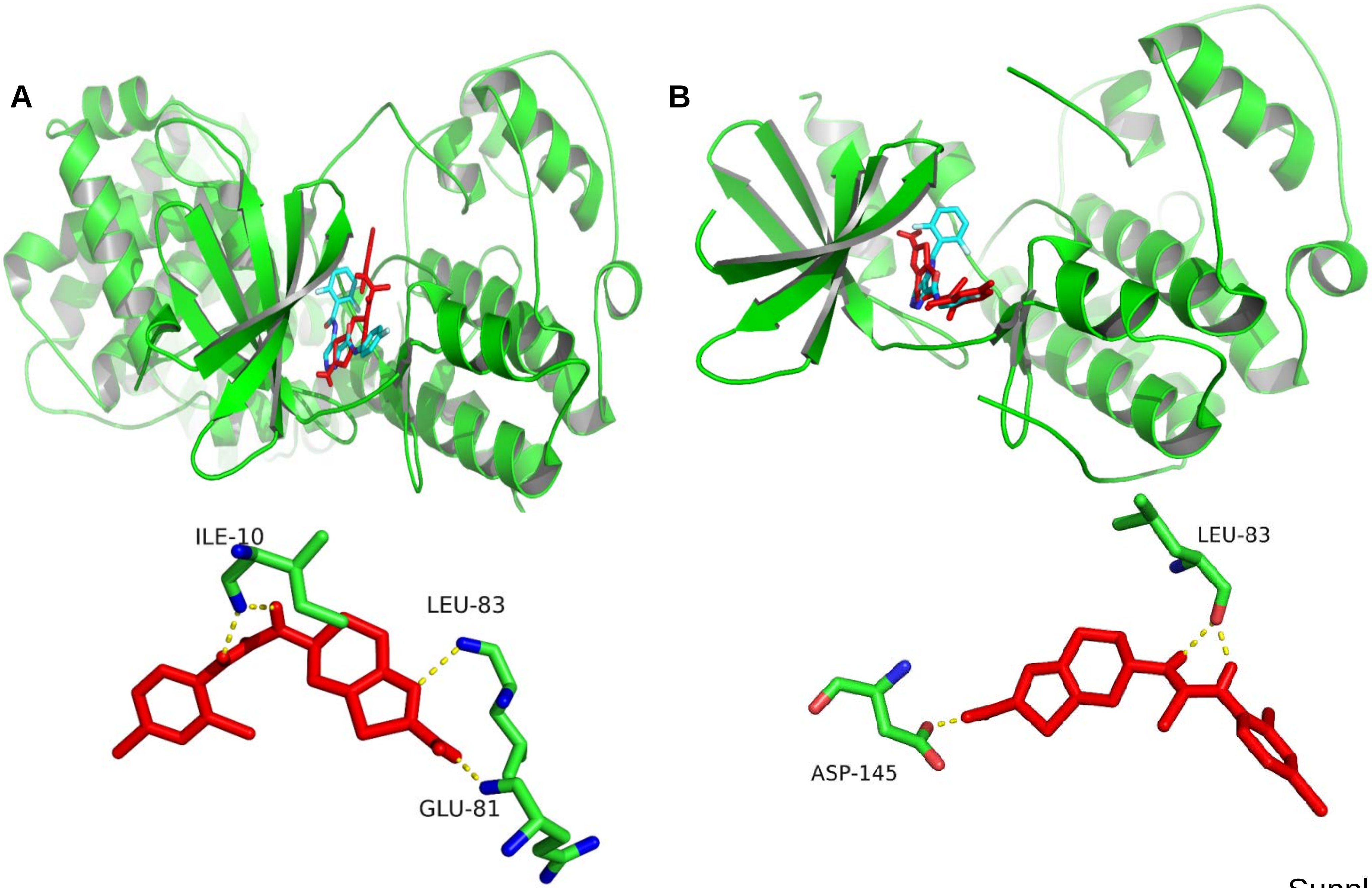


Suppl. Figure 4
